# Feasibility and safety of shared EEG/EOG and vision-guided autonomous whole-arm exoskeleton control to perform activities of daily living

**DOI:** 10.1038/s41598-018-29091-5

**Published:** 2018-07-17

**Authors:** Simona Crea, Marius Nann, Emilio Trigili, Francesca Cordella, Andrea Baldoni, Francisco Javier Badesa, José Maria Catalán, Loredana Zollo, Nicola Vitiello, Nicolas Garcia Aracil, Surjo R. Soekadar

**Affiliations:** 1 The BioRobotics Institute, Scuola Superiore Sant’Anna, Pisa, Italy; 2 Fondazione Don Carlo Gnocchi, Milan, Italy; 3 Applied Neurotechnology Laboratory, Department of Psychiatry and Psychotherapy, University Hospital of Tübingen, Tübingen, Germany; 4 Unit of Biomedical Robotics and Biomicrosystems, University Campus Bio-Medico of Rome, Rome, Italy; 5 Applied Robotics, Departamento de Ingeniería en Automática, Electrónica, Arquitectura y Redes de Computadores, Universidad de Cádiz, Cádiz, Spain; 6 Biomedical Neuroengineering, Departamento de Ingenieria de Sistemas y Automática, Universidad Miguel Hernandez de Elche, Elche, Spain; 7 Clinical Neurotechnology Laboratory, Neuroscience Research Center (NWFZ) & Department of Psychiatry and Psychotherapy, Charité – University Medicine Berlin, Berlin, Germany

## Abstract

Arm and finger paralysis, e.g. due to brain stem stroke, often results in the inability to perform activities of daily living (ADLs) such as eating and drinking. Recently, it was shown that a hybrid electroencephalography/electrooculography (EEG/EOG) brain/neural hand exoskeleton can restore hand function to quadriplegics, but it was unknown whether such control paradigm can be also used for fluent, reliable and safe operation of a semi-autonomous whole-arm exoskeleton restoring ADLs. To test this, seven abled-bodied participants (seven right-handed males, mean age 30 ± 8 years) were instructed to use an EEG/EOG-controlled whole-arm exoskeleton attached to their right arm to perform a drinking task comprising multiple sub-tasks (reaching, grasping, drinking, moving back and releasing a cup). Fluent and reliable control was defined as average ‘time to initialize’ (TTI) execution of each sub-task below 3 s with successful initializations of at least 75% of sub-tasks within 5 s. During use of the system, no undesired side effects were reported. All participants were able to fluently and reliably control the vision-guided autonomous whole-arm exoskeleton (average TTI 2.12 ± 0.78 s across modalities with 75% successful initializations reached at 1.9 s for EOG and 4.1 s for EEG control) paving the way for restoring ADLs in severe arm and hand paralysis.

## Introduction

Arm and hand paralysis due to lesions of the central or peripheral nervous system is the most common reason for long-term disability in the adulthood^1^. Particularly high-cervical spinal cord injuries, stroke or plexus brachialis avulsions resulting in a complete loss of arm and finger function have a substantial impact on the ability to perform various activities of daily living (ADLs), e.g. eating and drinking independently^2,3^.

Over the last years, various upper-limb robotic systems were developed to mobilize the upper limb and fingers, e.g. in the context of rehabilitation therapies^4–7^. Other promising robotic approaches to restore ADLs include gaze-based teleprosthetics^8^. While these systems were often immobile and designed to be used in rehabilitation facilities, recent advances in systems integration yielded the development of portable robotic arms with grippers^9,10^ or lightweight whole-arm^11^ or hand exoskeletons^12,13^ that can be used in everyday life environments to assist in ADLs. While assistive robotic arms were mainly designed for individuals with complete tetraplegia and inability to move their arms due to severe osteoporosis, atrophy of muscles and contractions of connective tissue, whole-arm exoskeletons that actuate the paralyzed upper-limb are particularly appealing for individuals with some remaining, but severely compromised arm and shoulder function. In this context, upper-limb exoskeletons can be either passive, for arm gravity compensation^14^, or active, for more complex assistive strategies^15^.

The main challenge to integrate such systems into everyday life environments relates to the individualization and user-friendliness of the hardware on one side, and the versatility, reliability and safety of robotic arm or exoskeleton control on the other side. Currently, there is no established paradigm for whole-arm exoskeleton control that is intuitive, safe and effective.

A very promising approach to provide intuitive control is based on direct translation of movement-related brain activity into robotic arm or exoskeleton control commands. The most impressive demonstrations of such approach required, however, the implantation of electrode-grids^16^ or microelectrode arrays^17^ with the risk of bleedings or infections.

Recently it was demonstrated that also non-invasive brain/neural recordings, such as electroencephalography (EEG) and electrooculography (EOG), allowed for intuitive and reliable hand exoskeleton control enabling quadriplegic patients with complete finger paralysis e.g. to eat and drink independently outside the laboratory^18^. In this study, EEG signal modulations related to the intention to grasp were translated online into actual grasping motions driven by a motorized hand exoskeleton system integrated into the users’ wheelchair. Hand exoskeleton opening motions as well as interruption of unintended hand closing motions were controlled by EOG signals related to voluntary horizontal oculoversions (HOV). While the majority of established control paradigms translating non-stationary and non-linear brain activity into control signals use a dual state approach (i.e. synchronous mode of operation)^19^, the applied system allowed for *asynchronous* mode of operation, i.e. end-users could initiate hand grasping motions at any time^18^.

In contrast to a simple grasping task, operating a whole-arm exoskeleton, for example to drink, involves a series of sub-tasks such as reaching, grasping and lifting. A seven degrees-of-freedom (DOF) system like the human arm in which each joint can assume e.g. three discrete joint positions would result in an actions space with a dimensionality of 2187 (3^7^). The necessity to increase the number of possible joint positions for fine finger control, as required to e.g. drink from a cup, would exponentially increase the dimensionality. Currently, information transfer rates (ITR) required for such high-dimensional control of a robotic whole-arm exoskeleton exceeds ITRs of any established (implantable or non-invasive) brain-machine interface (BMI) system. Thus, blending of BMI and e.g. vision-guided autonomous robotics was suggested to improve control performance^20^.

However, feasibility and safety of such novel control paradigm to restore ADLs, e.g. drinking, was not demonstrated yet. Here we tested whether an EEG/EOG control paradigm used to operate a vision-guided autonomous whole-arm exoskeleton across a series of sub-tasks (reaching, grasping, lifting and drinking, as well as placing back and releasing a cup) is feasible and safe.

**Figure 1:**
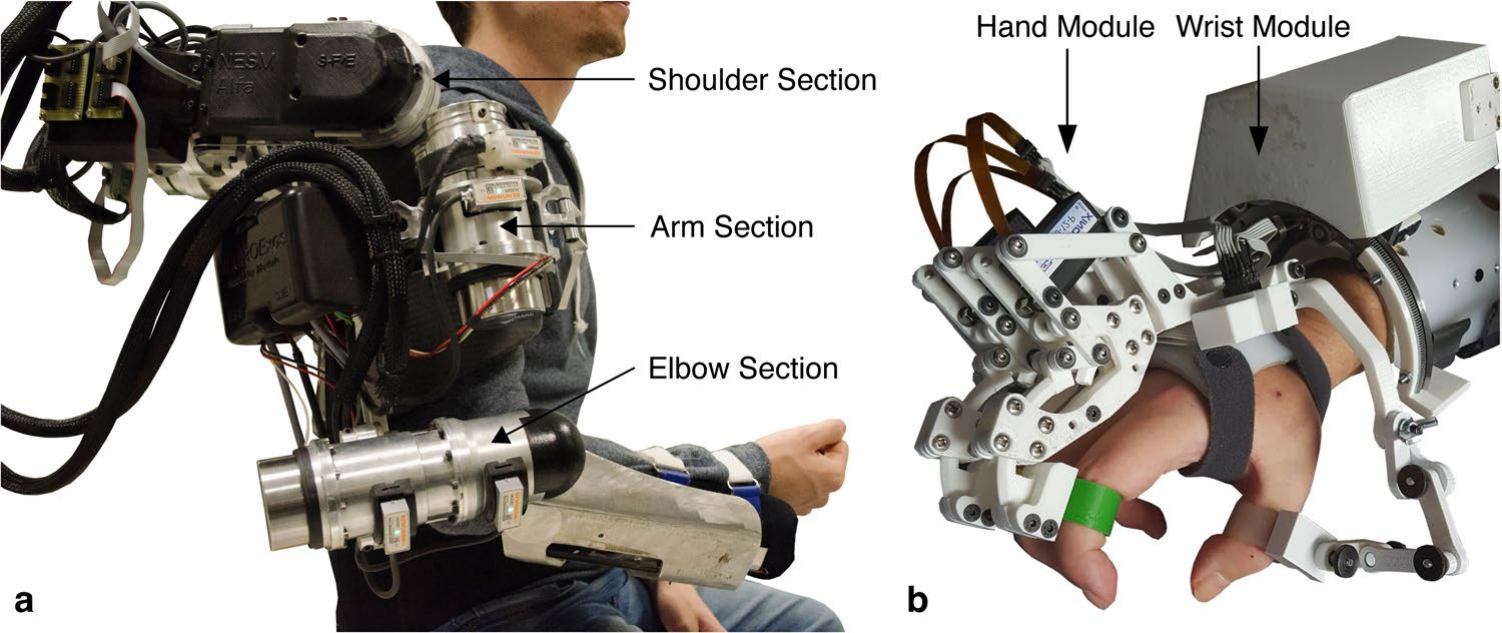
Illustration of the different components of the whole-arm exoskeleton. **(a)** NeuroExos Shoulder-elbow Module (NESM) exoskeleton consisting of three sections: shoulder, arm and elbow. **(b)** Hand-wrist exoskeleton comprising two modules: the hand module allows hand opening or closing motions, while the wrist module allows for pronation or supination movements.

**Figure 2:**
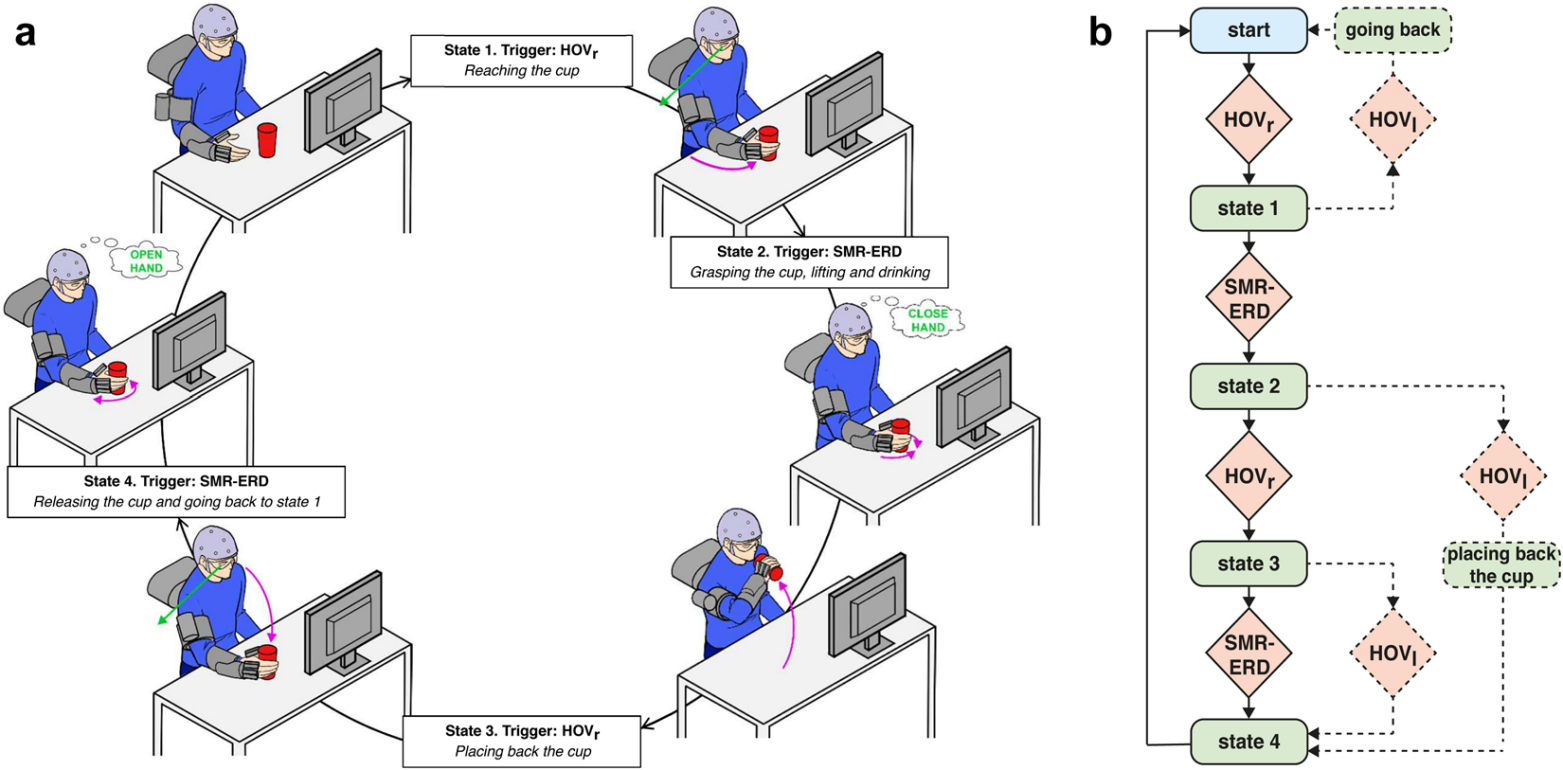
Shared-human robot control strategy based on a finite-state machine (FSM) triggered by electroencephalography/electrooculography (EEG/EOG). **(a)** Visualization of whole-arm exoskeleton actions controlled by EEG/EOG. Green arrows indicate horizontal oculoversions to the right (HOV_r_) recorded with EOG, while “close hand” and “open hand” indicate EEG desynchronization of sensorimotor rhythms (SMR-ERD, 9–15 Hz) related to motor imagery of grasping and releasing motions. Purple arrows represent actions of the whole-arm exoskeleton. (Drawings: D. Marconi, The BioRobotics Institute, Scuola Superiore Sant’Anna, Pisa, Italy). **(b)** Flowchart of the whole-arm exoskeleton control loop.

While hand opening and closing motions to grasp and release a cup were controlled by motor imagery-related EEG desynchronization of sensorimotor rhythms (SMR, 9–15 Hz), reaching, lifting and placing back of the cup were controlled by EOG signals related to horizontal oculoversions (HOV) to the right (HOV_r_). At any time, the participants could re-set the system to the initial state by using HOV to the left (HOV_l_) (Figs 1 and 2). The movement trajectories of the whole-arm exoskeleton were continuously calculated and adapted using optical object tracking. Fluency, defined as average ‘time to initialize’ (TTI) execution of each of the sub-tasks, and reliability, defined as time to successfully initialize 75% of sub-tasks, as well as safety of operation were assessed. We reasoned that an average TTI of more than 3 seconds across control modalities would not be conceived as fluent operation, given that temporal integration in human sensorimotor control occurs in a 3-second time window^21,22^. Reliable control was assumed if 75% of sub-tasks were successfully initialized within a time window of 5 seconds, a value comparable to other state-of-the-art BMI systems using an asynchronous mode of operation for exoskeleton^18,23^. Additionally, EEG/EOG control performance was assessed by calculating the sensitivity index (SI)^23^ that reflects the average rate of false positive classifications. At the end of the sessions, all participants were asked to report any discomforts or undesired side-effects.

## Results

### Feasibility

**Figure 3:**
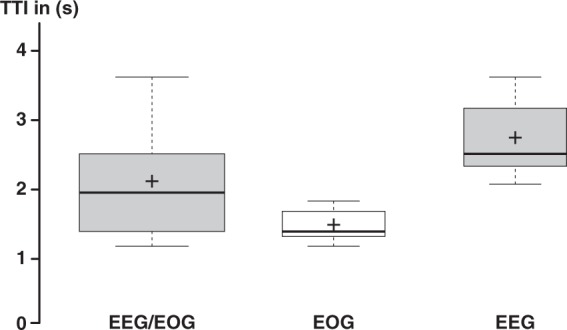
Fluency. ‘Time to initialize’ (TTI) across control modalities (EEG/EOG) as well as for individual EOG and EEG control mode across all participants. Average TTIs ranged below 3 s documenting fluent control. Crosses show the means, while centrelines show the medians. Box limits indicate the 25th and 75th percentiles.

The average TTI (±s.d.) across control modalities ranged at 2.12 ± 0.78 s (median = 1.96 s [interquartile range = 1.46–2.48 s]) documenting fluent whole-arm exoskeleton control. The average EOG TTI was 1.49 ± 0.25 s (median = 1.40 s [1.32–1.69 s]), while average EEG TTI ranged at 2.75 ± 0.57 s (median = 2.52 s [2.34–3.18 s]) (Fig. 3).

**Figure 4:**
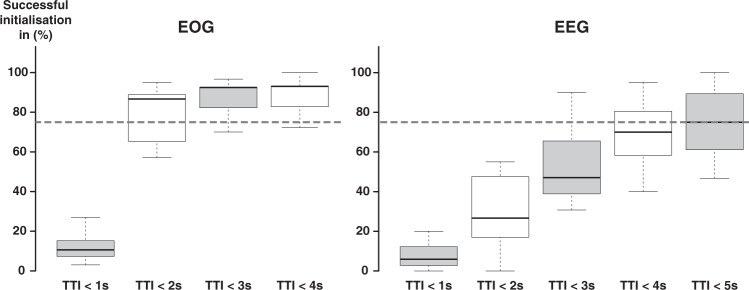
Reliability. Successful initializations during EEG and EOG control for discrete time intervals. Box plots show the relative number of successful initializations with TTIs smaller or equal to discrete time intervals ranging from 1 to 5 s for EEG and 1 to 4 s for EOG. Dashed line indicates 75% threshold of successful initializations representing reliable control, which was assumed when the time for successful initializations ranged below 5 s. The exact time to initialize 75% of the EOG-controlled sub-tasks was 1.9 s, while time to initialize 75% of the EEG-controlled sub-tasks was 4.1 s. Centrelines show the medians. Box limits indicate the 25th and 75th percentiles.

Time to initialize 75% of the EOG-controlled sub-tasks was 1.9 s, while time to initialize 75% of the EEG-controlled sub-tasks was 4.1 s. Thus, time for 75% of successful initializations ranged below 5 s documenting reliable control (Fig. 4).

### Safety

At the end of the session, none of the participants reported any discomforts or side-effects during whole-arm exoskeleton control.

### Control performance

All participants were able to control the whole-arm exoskeleton using EEG/EOG control. SI (±s.d.) reached an average value of 1.43 ± 0.62 for EEG and 3.63 ± 1.21 for EOG across participants indicating good separation of EEG and EOG mean power values during rest and task and low false positive rate during use of the system (see classification rates, Table 1).

**Table 1:** Hit and false alarm classification rates across control modalities.

	EEG	EOG
Hit rate in % (±s.d.)	64.7 ± 5.0	94.3 ± 7.3
False alarm rate in % (±s.d.)	17.0 ± 21.4	5.7 ± 7.3

## Discussion

Various neurological disorders related to brain lesions, neurodegeneration or neuroinflammation can lead to complete loss of hand and arm function. To restore this function, we developed a whole-arm exoskeleton^24^ and tested whether a novel shared human-robot control strategy based on EEG/EOG signals can be used for its fluent and reliable operation, e.g. to reach out, lift and drink from a cup. While average TTIs during EOG control required less than 2 seconds (1.49 ± 0.25 s), brain control was associated with longer TTIs (2.75 ± 0.57 s), but still ranged below 3 seconds. This longer duration before initialization may relate to the time required for task-switching. Moreover, the delay in maximum ERD usually ranging between 800 milliseconds and up to 2 seconds^18,25^. Performance of EEG/EOG control during use of this novel shared human-robot control strategy was comparable to other EEG/EOG control paradigms, e.g. asynchronous operation of a brain/neural hand exoskeleton^23^ or a robotic arm^26^. None of the participants complained about any discomfort or undesirable effects. Clinical studies are needed that investigate whether these findings can be generalized to individuals suffering from severe arm and hand paralysis.

While using EEG alone to control a complex multi-joint exoskeleton would be impractical due to the limited bandwidth and reliability, combined EEG and electromyography (EMG) control would provide higher bandwidth and reliability. However, because most people who require an exoskeleton suffer from a neurological condition that affects muscle control (often resulting in early fatigue), reliable EMG control often requires precise placing of the electrodes, extensive training and repeated individual calibration. Besides EOG, another viable tool for shared brain/neural- and vision-guided autonomous robot control might be eye tracking, an approach worth further exploring. Particularly, in more complex tasks with many branches, predominant use of EOG or eye tracking might be appealing. Besides using the system to perform a drinking task, it is conceivable that also other ADLs can be restored. In such case, the different tasks could be selected using sensor glasses to initiate different movement trajectories depending on the user’s gaze direction.

Before whole-arm exoskeletons can enter everyday life environments, some technical challenges need to be addressed. These challenges relate to their weight, mobility and adaptability to the specific anatomical characteristics of possible end-users. Especially the presence of a stationary camera limits the portability of the current system. Portability could be improved by integrating the camera (possibly a set of cameras) into the wheelchair. Another challenge relates to the safety of such systems: while our study was performed in a controlled lab environment, further studies are needed to investigate possible safety constraints in everyday life scenarios. In this respect, implementation of a reliable veto function^27^, i.e. the ability to interrupt unintended motions or behaviours of the system, will be critical. In our setup, the user could re-set the system to the initial state by eliciting HOV to the left (HOV_l_). Such veto function will be required for any brain-controlled device, as none of the existing BMI approaches provide sufficient reliability in decoding brain activity to exclude false positive classifications (ranging at 17.0 ± 21.4% in our control paradigm with an asynchronous mode of operation, Table 1). Future studies should investigate the practicality and reliability of different veto-mechanisms across various everyday life scenarios, an issue not investigated in our study.

While voluntary muscle contractions have a “point of no return” approximately 200 milliseconds before their execution^28,29^, voluntary acts can still be modified or vetoed after this “point of no return”. However, such “last minute” modification cannot be provided by any means of a BMI system, as the time window for detection of a false positive classification and corresponding brain response (usually a P300, i.e. a positive EEG deflection approximately 300 ms after error detection) would exceed this period. It is conceivable, however, that once the neural substrate of vetoing was identified, a veto function based on brain activity classification is feasible.

Previous studies indicated that the majority of individuals with damaged brain areas, e.g. following a stroke, can successfully use a brain-machine interface (BMI) to open and close a hand-orthosis^30–32^. Whereas in healthy volunteers, EEG signals from electrode positions C3 or C4 (located over the motor cortex) are usually used, electrode positioning for BMI control may need to be adapted after a brain lesion due to cortical reorganization^33^. Given the growing evidence that repeated use of a brain-controlled exoskeleton can trigger neurological recovery^34,35^, larger studies that also investigate this aspect are needed^36^. Currently, there is no study that investigated whether shared EEG/EOG and vision-guided autonomous whole-arm exoskeleton control would result in similar neurorehabilitation effects as EEG exoskeleton control alone. In this context, characterizing the optimal training schedules for specific patient populations will be important. Implementation of other bio-signals into shared human-robot interaction, such as physiological measures reflecting the user’s workload or mental state, e.g. heart-rate variability^37^, might improve their applicability and provide important information to optimize training schedules in the context of neurorehabilitation. By demonstrating feasibility and safety of shared EEG/EOG and vision-guided autonomous whole-arm exoskeleton control as accomplished in our study, such further clinical testing is now rendered possible.

## Materials and Methods

### Participants

Seven healthy volunteers (seven males, mean age (±s.d.): 30 ± 8 years) were invited to a single-session experiment at the Università Campus Biomedico di Roma (Rome, Italy). Before entering the study, all participants provided written informed consent. To enter the study, the following inclusion criteria had to be met: (i) absence of neurological or physical disorders, (ii) no regular medication intake, (iii) no history of neurological or psychiatric disorders and (iv) ability to speak and understand Italian or English. The study protocol complied with the Declaration of Helsinki and was approved by the local ethics committee (Comitato Etico Università Campus Biomedico di Roma, reference number: 01/17 PAR ComEt CBM) and by the Italian Ministry of Health (Registro - classif. DGDMF/I.5.i.m.2/2016/1096).

### Whole-arm exoskeleton

The whole-arm exoskeleton comprises two components: A shoulder-elbow exoskeleton and a hand-wrist exoskeleton.

#### Shoulder-elbow exoskeleton

The shoulder-elbow exoskeleton (NeuroExos Shoulder-elbow Module, NESM) is a robotic upper-limb exoskeleton for shoulder/elbow mobilization^24,38^ (Fig. 1a). The exoskeleton was hanging from a standing structure and provides four active DOFs that are all rotational joints mounted in a serial kinematic chain. The exoskeleton mechanical structure is composed of three main blocks: the shoulder section, the arm section and the elbow section. The shoulder section includes the actuation units and motor drivers for the shoulder adduction/abduction (sA/A) and flexion/extension (sF/E). Both the sA/A and sF/E actuation units can exert a maximum continuous torque of 60 N∙m. The arm section includes a shoulder intra/extra (sI/E) rotation joint. The actuation unit can exert a maximum continuous torque of 30 N∙m. Moreover, the arm support consists of carbon-fiber shell structures with aluminium inserts and an ergonomic cuff. Finally, the elbow section includes an elbow flexion/extension (eF/E) joint. This joint can exert a maximum torque of 30 N∙m.

Each actuation unit has a series elastic actuation (SEA) architecture comparable to Vitiello, *et al*.^39^ that includes the following components: A brushless direct current (DC) motor, a reduction stage, two absolute encoders, a custom spring and an Elmo® Gold Solo Whistle servo driver. In each joint, the two encoders are placed at the two sides of the spring with respect to the mechanical frame: the encoder more proximal to the human joint (i.e. the joint position encoder) provides the joint angular value, while the difference between the two encoder readings provides the spring deformation, thus the transmitted torque. The support structure of the exoskeleton consists of a wheeled platform endowed with a vertical stand, which carries an articulated parallelogram functioning as a weight-relief system and supports the exoskeleton through a passive rotational joint. The wheeled platform also hosts the box containing the control and interface electronics. The total weight of the device (with the support structure, counterweight and electronics) was around 136 kg whereas the only wearable exoskeleton module weights approximately 13 kg. Passive DOFs allowing the rotation axis to follow the axis of the corresponding biological joint, and size regulations allowing to adjust the lengths of the frames to the user’s anatomical characteristics, have been designed to ensure user comfort.

The control system was implemented on a real-time controller, a sbRIO-9636 (National Instruments, Austin, Texas, US) endowed with a 400-MHz processor running a NI real-time operating system and a field programmable gate array (FPGA) processor Xilinx Spartan-3. While the high-level layer was running at 100 Hz, the low-level closed-loop controllers were running at 1 kHz.

On the low-level control layer, a joint position control mode was implemented. Each actuation unit drives the joint position along a reference value or trajectory. The closed-loop joint position control of each actuation unit employed a proportional-integrative-derivative (PID) regulator using the error between the desired joint angle and measured joint position to allow for passive guidance in the absence of residual movement capabilities.

#### Hand-wrist exoskeleton

The hand-wrist exoskeleton consists of two modules: The hand and wrist module that can be used separately or in combination^40^ (Fig. 1b).

The hand exoskeleton was an electrical-driven device with 4-active DOFs. The mechanical structure of the exoskeleton can be divided into three main parts: (1) First and second fingers: For each of these fingers, a linkage attached to proximal and middle phalanxes is driven by a linear actuator, performing a movement of both proximal interphalangeal (PIP) and metacarpophalangeal (MCP) joints. (2) Third and fourth fingers: A similar linkage is used for the third finger, while the linkage for the fourth finger is only attached to the proximal phalanx. Both linkages are driven by the same linear actuator, performing a movement of the third finger’s PIP and MCP and fourth finger’s MCP. (3) Thumb: For the thumb only, the flexion/extension movement is actuated, while adduction/abduction movement is fixed in an abduction position in order to achieve thumb opposition. In the same way as the fourth finger, a linear actuator drove a linkage to perform the movement of the thumb.

The wrist exoskeleton was a 1-active DOF device for the mobilization of the pronation/supination movement of the wrist. It consisted of a direct current (DC) motor with a reduction stage that drove a geared ring guide. The circular guide was attached to an orthosis that aligned the forearm with the axis of the guide. In addition, two load cells were used to estimate the interaction forces between the device and the user and ensured a safe human-robot interaction.

The wrist exoskeleton was designed to be easily connected to the NESM exoskeleton: by simply removing the forearm cuff from the NESM, the cuff integrated to the wrist exoskeleton could be attached to the elbow actuation unit’s output frame.

### EEG/EOG control interface

A 5-channel, wireless EEG (Enobio^®^, Neuroelectrics Barcelona S.L., Spain) was recorded from the following conventional 10/20 system recording sites: F3, T3, C3, Cz and P3 using polyamide-based solid-gel electrodes^41^. Ground and reference electrodes were placed at AFz and FCz, respectively. One additional channel was used to detect HOV using EOG signals recorded from the left outer canthus referenced to left mastoid. EEG and EOG were sampled at 500 Hz and band pass-filtered at 0.1–30 Hz. To increase signal-to-noise ratio, EEG was pre-processed using a surface Laplacian filter^42^. A customized version of the open-source BCI2000 software^18,43^ was used to translate the EEG and EOG signals into whole-arm exoskeleton control commands. To compute SMR event-related desynchronization (SMR-ERD), the power method by Pfurtscheller and Lopes da Silva^44^ was used.

### Motion capturing system

To capture the position and motion of the whole-arm exoskeleton and the graspable object (e.g. a cup) allowing vision-guided control, an IR-camera was used (OptiTrack^®^, Corvallis, USA). The system was placed on a vertical stand and tracked objects in 6 DOFs using reflective markers that were attached to the graspable object.

### System component communication

**Figure 5:**
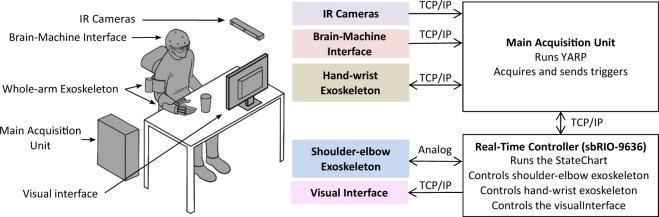
Overview of components and communication architecture based on TCP/IP protocol and analogue communication. (Drawings: D. Marconi, The BioRobotics Institute, Scuola Superiore Sant’Anna, Pisa, Italy).

The communication among all the integrated components, i.e. the whole-arm exoskeleton, EEG/EOG control interface and motion capturing system, was managed by the messaging system YARP (Yet Another Robotic Platform)^45,46^. YARP external nodes were created in C++, MATLAB, LabVIEW and Linux environment for sending and receiving data to and from other nodes via a TCP/IP connection. Each node receiving and sending data was identified by a label and had a unique communication port on the YARP server. For the study, all data were received and transmitted at a frequency of 20 Hz. After starting the YARP server, all other nodes could connect independently and, if a node suddenly disconnected, the others could continue sending and receiving data without jeopardizing communication. The scheme of the communication architecture is given in Fig. 5.

### Experimental setup and protocol

All participants were comfortably seated at a desk and equipped with a whole-arm exoskeleton as well as wearable devices including EEG and EOG. Before any calibration procedures, participants were familiarized with each of the components and received detailed instructions about the sequence of the drinking task. The drinking task comprised the following sub-tasks: 1. reaching a cup, 2. grasping and drinking from the cup, 3. placing back the cup and 4. releasing the cup. To evaluate fluency and reliability of EEG/EOG control, participants received visual instructions when to execute each of the sub-tasks. While sub-tasks 1 and 3 were initialized by EOG signals, sub-tasks 2 and 4 were controlled by EEG signals. Over all, each participant performed the whole drinking task 20 times (i.e. 80 sub-tasks in total).

After detailed instruction, all devices for EEG/EOG assessment including amplifier and electrode cap were mounted. The EEG/EOG control interface was calibrated at the beginning of the session and remained unchanged for the entire session. During calibration, detection thresholds for SMR-ERD and HOV were determined as in Soekadar, *et al*.^18^. For calibration of the EEG/EOG control interface, a reference value (RV) of SMR-ERD related to externally-paced imagined hand opening or closing movements of the right hand was calculated by using a power spectrum estimation based on an autoregressive model of order 16 (Burg algorithm). Calculation of the RV comprised a total of 42 trials, each lasting 5 s, followed by an inter-trial interval (ITI) of 4 s, during which participants were inactive (rest condition). The optimal frequency for SMR-ERD detection [frequency of interest (FOI)] was set to the frequency showing the highest SMR-ERD modulation during imagined movements. For online classification of SMR-ERD, a frequency filter with an FOI of ±1.5 Hz was used. A detection threshold for movement initiation and execution was calculated on the basis of the additional 42 trials, during which participants received online visual feedback of SMR-ERD provided on the display in front of them. The detection threshold was set at two standard deviations (s.d.) above average SMR-ERD and used for online EEG control. SMR-ERD was translated into a control command if detection threshold was exceeded. As a next step, participants were instructed to perform 10 externally paced HOV to the left or right following a visual cue while EOG was recorded. In analogy to EEG calibration, a HOV detection threshold was set at two s.d. below the average EOG signal recorded during maximum HOV. While HOV to the right (HOV_r_) was defined as confirmatory signal in states 1 and 3, the user could re-set the system to the initial state at any time by HOV to the left (HOV_l_) (Fig. 2). Mounting and calibration of the EEG/EOG control interface required approximately 15–20 minutes per participant. Since there was just one session per participant, the whole donning and calibration procedure was performed only once at the beginning of the session.

Before performing 20 trials with the whole-arm exoskeleton to drink from a glass, participants were familiarized with wearing and moving the whole-arm exoskeleton by performing one complete drinking sequence.

### Shared human-robot control

To operate the multi-DOF whole-arm exoskeleton, a shared-human robot control strategy was used based on a finite-state machine (FSM) blending vision guidance and BMI control. FSM was implemented within the 100 Hz main loop of the NESM high-level control layer. The following states were implemented in the FSM (Fig. 2):

State 1. *Reaching the cup*: Detection of HOV results in a whole-arm exoskeleton reaching movement guided by the motion capturing system.

State 2. *Grasping the cup, lifting and drinking*: Detection of SMR-ERD exceeding the SMR-ERD detection threshold results in exoskeleton-driven hand closing motions. Once the grasping motion is complete, the whole-arm exoskeleton automatically lifts the cup and moves it to the user’s mouth without any additional trigger elicited by the participant.

State 3. *Placing back the cup*: After drinking from the cup, detection of HOV results in moving the whole-arm exoskeleton back to the position from where the cup was lifted.

State 4. *Releasing the cup and going back to state 1*: Detection of SMR-ERD results in exoskeleton-driven hand opening motions. Once the grasping motion is complete, the whole-arm exoskeleton automatically moves back to state 1, without any additional trigger.

An inverse kinematics algorithm calculated the joint angles to achieve the desired end-effector position as described in Lauretti, *et al*.^47^. The end-effector moves along a linear 3D trajectory, as each joint moves along a pre-defined trajectory with a stereotyped sigmoid time-position profile, executed within 5 s. Moreover, the exoskeleton end-effector position was finely adjusted via a *visual-servoing* algorithm: the error between the end-effector and object position was calculated online. End-effector positions were corrected until the error dropped below a certain threshold (i.e. 2 cm).

A graphical user interface (GUI) allowed to visualize the inputs and outputs of the FSM as well as communication flow between the different system components. Moreover, a user display was used to provide the following visual cues that were synchronized with the system’s FSM state:

1. EOG control 1: “Please look to the right to initialize reaching movement.” (State 1)
2. EEG control 1: “Cup reached! Please imagine hand closing to grasp the cup.” (State 2)
3. EOG control 2: “Please look to the right for placing back the cup!” (State 3)
4. EEG control 2: “Initial cup position reached! Please imagine hand opening to release the cup.” (State 4)

A green square indicated when the sub-task was successfully initialized.

### Data collection and analyses

During use of the system, all system parameters as well as physiological data were stored for offline analysis. EEG/EOG TTIs were evaluated as time between visual indication and detection of EEG/EOG signals exceeding the EEG/EOG detection thresholds. Reliability of control was assessed by calculating the time for successful initializations of at least 75% of sub-tasks. Reliable control was assumed when the time for successful initializations ranged below 5 s. To evaluate the average rate of false positive EEG/EOG classifications as measure of control performance, the sensitivity index (SI, Eq. 1) was calculated and averaged across all participants. SI of EEG and EOG, respectively, was assessed based on 20 trials, which were recorded at the end of the calibration phase.

1 $${\rm{d}}^{\prime} ={\rm{Z}}({\rm{hit}}\_{\rm{rate}})\mbox{--}{\rm{Z}}({\rm{false}}\_{\rm{alarm}}\_{\rm{rate}})$$

For EEG, ‘hit_rate’ was defined as the relative number of sample blocks during the task in which EEG activity exceeded the EEG-detection threshold, whereas ‘false_alarm_rate’ was defined as the relative number of sample blocks during rest in which the EEG-detection threshold was exceeded. For EOG, a ‘hit’ (true positive) was assumed if EOG signals exceeded the EOG-detection threshold upon visual instruction, whereas ‘false_alarm’ (false positive) was assumed if EOG signals exceeded the EOG-detection threshold although no visual instruction was provided.

### Data availability

The datasets generated during and/or analysed during the current study are available from the corresponding author on reasonable request.
